# Impact of calorie labelling in worksite cafeterias: a stepped wedge randomised controlled pilot trial

**DOI:** 10.1186/s12966-018-0671-7

**Published:** 2018-05-14

**Authors:** Milica Vasiljevic, Emma Cartwright, Mark Pilling, Mei-Man Lee, Giacomo Bignardi, Rachel Pechey, Gareth J. Hollands, Susan A. Jebb, Theresa M. Marteau

**Affiliations:** 1 Behaviour and Health Research Unit, Institute of Public Health, University of Cambridge, Cambridge, CB2 0SR UK; 2 Nuffield Department of Primary Care Health Sciences, University of Oxford, Oxford, UK

**Keywords:** Physical micro-environment, Choice architecture, Nudging, Stepped wedge trial, Randomised controlled trial, Healthier eating, Workplace interventions, Calorie labelling

## Abstract

**Background:**

For working adults, about one-third of energy is consumed in the workplace making this an important context in which to reduce energy intake to tackle obesity. The aims of the current study were first, to identify barriers to the feasibility and acceptability of implementing calorie labelling in preparation for a larger trial, and second, to estimate the potential impact of calorie labelling on energy purchased in worksite cafeterias.

**Methods:**

Six worksite cafeterias were randomised to the intervention starting at one of six fortnightly periods, using a stepped wedge design. The trial was conducted between August and December 2016, across 17 study weeks. The intervention comprised labelling all cafeteria products for which such information was available with their calorie content (e.g. “250 Calories”) displayed in the same font style and size as for price. A post-intervention survey with cafeteria patrons and interviews with managers and caterers were used to assess the feasibility and acceptability of the intervention. Intervention impact was assessed using generalised linear mixed modelling. The primary outcome was the total energy (kcal) purchased from intervention items in each cafeteria each day.

**Results:**

Recruitment and retention of worksite cafeterias proved feasible, with post-intervention feedback suggesting high levels of intervention acceptability. Several barriers to intervention implementation were identified, including chefs’ discretion at implementing recipes and the manual recording of sales data. There was no overall effect of the intervention: -0.4% (95%CI -3.8 to 2.9, *p* = .803). One site showed a statistically significant effect of the intervention, with an estimated 6.6% reduction (95%CI -12.9 to − 0.3, *p* = .044) in energy purchased in the day following the introduction of calorie labelling, an effect that diminished over time. The remaining five sites did not show robust changes in energy purchased when calorie labelling was introduced.

**Conclusions:**

A calorie labelling intervention was acceptable to both cafeteria operators and customers. The predicted effect of labelling to reduce energy purchased was only evident at one out of six sites studied. Before progressing to a full trial, the calorie labelling intervention needs to be optimised, and a number of operational issues resolved.

**Trial registration:**

ISRCTN52923504; Registered: 22/09/2016; retrospectively registered.

**Electronic supplementary material:**

The online version of this article (10.1186/s12966-018-0671-7) contains supplementary material, which is available to authorized users.

## Background

In the UK context, poor diets are the leading modifiable risk factor for excess mortality [1]. Encouraging people to make healthier food choices whilst reducing excess consumption of food and drink is therefore core to improving health outcomes in the population [2]. Current estimates suggest that about one-third of a working adult’s daily energy intake is consumed whilst at work [3], making the workplace a potentially important setting for dietary interventions. To date, evidence regarding this potential is limited in quantity and quality.

A systematic review examining workplace dietary interventions suggested that workplace interventions may lead to small increases in fruit and vegetable consumption [4]. However, this review was based on four studies using self-reported outcome measures of fruit and vegetable consumption, following interventions that were mainly information-based (including different kinds of nutritional education campaigns communicated using posters, leaflets, and group workshops delivered at workplaces). Current evidence suggests that information-based interventions that rely on people’s conscious engagement with the material presented are most often insufficient to change the routine and habitual behaviours characteristic of food selection and consumption [5]. More promising for behavior change are interventions that involve altering cues in physical micro-environments that are proximal to and shape much of our behaviour, often without awareness [6, 7]. Such interventions are often referred to as ‘choice architecture’ or nudging interventions [8].

In line with the above, a recent systematic review examining choice-architecture dietary interventions in the workplace found that such interventions have the potential to increase fruit and vegetable consumption, increase sales of healthy options, and reduce total calories purchased [9]. Despite the promising findings, the multi-component nature of many of the interventions precluded isolating the effectiveness of individual interventions and the frequent use of self-report measures of purchasing undermined confidence in the validity of the results of many of the studies. The authors concluded that larger trials using designs that can isolate the effects of individual interventions are needed to ascertain whether the promise of choice architecture interventions in worksite cafeterias can be realised.

Labelling is one intervention for reducing consumption of food which was deployed in some of the choice-architecture intervention studies reviewed by Allan and colleagues [9]. The results of a recent Cochrane review of nutritional labelling indicate the promise of this approach, finding that calorie labelling at point of consumption in restaurant settings has the potential to reduce average daily energy intake from food and drinks by an estimated 7.8% per meal (with a 95% confidence interval ranging from 2.5% to 13.1%) [10]. However, the synthesised effect size was based on limited evidence derived from three randomised controlled trials in US restaurant settings. Moreover, there were no studies in worksite cafeterias that met the review inclusion criteria. The present research aims to fill this gap by examining the impact of calorie labelling in worksite cafeterias and extending the reach of the labelling beyond menus (to include both product and shelf labelling).

This study is part of a pilot trial testing the impact of each of three physical micro-environment interventions - calorie labelling, portion size, and availability of healthier options - to reduce energy purchased in workplace cafeterias in preparation for a future larger trial (for the published protocol see [11]). The three interventions were implemented and evaluated separately, involving a total of 18 sites. We report here the results of the calorie labelling intervention, which involved labelling foods and non-alcoholic drinks with their energy (kcal) content in six worksite cafeterias.

The aims of the present study are:

1. to assess the feasibility of recruiting eligible worksites, and identify potential barriers to the feasibility and acceptability of implementing the labelling intervention; and
2. to estimate the potential impact of calorie labelling upon energy purchased.

## Methods

### Sample

Six English worksite cafeterias were recruited from the 1027 companies that are members of the Institute of Grocery Distribution (IGD) [12]. IGD is a charity set up to inform and educate the food and grocery industry about best practice. Worksites from any region within England were eligible. Selection criteria for study sites were as follows:

1. Site size: employing more than 350
2. Ability to provide at least weekly data on sales of individual items and their energy content

**Fig. 1:**
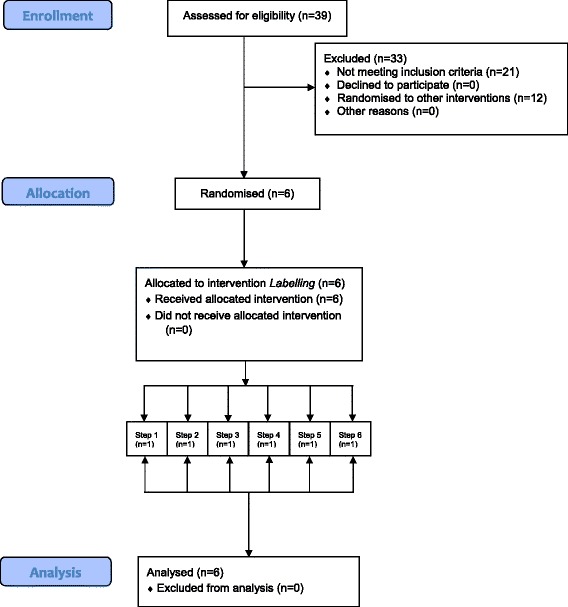
CONSORT diagram of participant flow through the study

We identified 39 sites that seemed likely to meet the two criteria, based on information available to IGD and contacted the managers of these sites. As such, our sampling frame included food and grocery industry worksites who wanted to encourage healthier eating amongst their workforce and supported the current research as part of this initiative. Twenty-one sites did not meet the criteria. Of the 18 that did, six were selected to implement the calorie labelling intervention (based on readiness to participate in the study, since this was the first intervention implemented), with the remaining 12 selected to implement, the availability and portion size interventions. Enrolment of sites into the study was completed by a research assistant (EC). Managers of participating worksites provided their consent for the cafeteria to be included in the present trial before the study period commenced. A CONSORT flow diagram delineating the flow of participating sites through the pilot trial is provided in Fig. 1.

The demographic characteristics of employees varied across the six sites (Table 1). For example, Site 6 mainly employed male semi-skilled or unskilled manual workers, whereas Sites 2 and 6 had a higher proportion of temporary seasonal workers when compared to the other sites. Furthermore, Site 1 was more than twice the size of Site 6, and Sites 1 and 2 employed a greater proportion of older skilled manual workers.

**Table 1:** Staff demographic characteristics and baseline sales data across the six sites

Categories	Site 1 (*n* = 1163)	Site 2 (*n* = 756)	Site 3 (*n* = 978)	Site 4 (*n* = 1031)	Site 5 (*n* = 750)	Site 6 (*n* = 530)
Employment Type [*n* (%)]						
Full Time	1070 (92%)	649 (56%)	904 (92%)	957 (93%)	525 (70%)	525 (52%)
Part Time	93 (8%)	106 (9%)	74 (8%)	74 (7%)	75 (10%)	5 (0.5%)
Temporary	0 (0%)	394 (34%)	0 (0%)	0 (0%)	150 (20%)	476 (47%)
Gender [*n* (%)]						
Male	709 (61%)	409 (54%)	393 (40%)	417 (40%)	375 (50%)	484 (91%)
Female	454 (39%)	348 (46%)	585 (60%)	614 (60%)	375 (50%)	36 (9%)
Age [*n* (%)]						
18–24	76 (6%)	42 (6%)	64 (7%)	96 (9%)	75 (10%)	42 (8%)
25–34	306 (26%)	103 (14%)	394 (40%)	325 (32%)	375 (50%)	95 (18%)
35–44	307 (26%)	126 (17%)	326 (33%)	316 (31%)	75 (10%)	104 (20%)
45–54	61 (5%)	260 (34%)	152 (16%)	204 (20%)	150 (20%)	175 (33%)
55–64	413 (36%)	214 (28%)	38 (4%)	89 (9%)	75 (10%)	11 (2%)
65+	0 (0%)	10 (1%)	0 (0%)	7 (0.6%)	0 (0%)	1 (0.2%)
Role Type [*n* (%)]						
Higher Managerial	65 (6%)	7 (1%)	89 (9%)	19 (2%)	75 (10%)	11 (2%)
Intermediate Managerial	300 (26%)	15 (2%)	374 (38%)	704 (68%)	300 (40%)	6 (1%)
Supervisory or Clerical/Junior Managerial	454 (39%)	64 (8%)	515 (53%)	289 (28%)	375 (50%)	58 (11%)
Skilled Manual Worker	344 (30%)	539 (71%)	0 (0%)	0 (0%)	0 (0%)	96 (18%)
Semi or Unskilled Manual Worker	0 (0%)	124 (16%)	0 (0%)	0 (0%)	0 (0%)	283 (54%)
Other	0 (0%)	6 (1%)	0 (0%)	12 (1%)	0 (0%)	30 (6%)
Sales Data at Baseline						
Number of Daily Transactions [Mean (SD)]	850 (99)	240 (37)	1099 (203)	1033 (211)	489 (71)	560 (86)
Main meal kcal [Mean (min, max)]	462 (118, 1444)	399 (135, 757)	455 (149, 1320)	526 (62, 837)	309 (106, 1131)	388 (135, 869)
Drink kcal [Mean (min, max)]	171 (0, 395)	158 (0, 260)	27 (0, 46)^a^	70 (0, 119)	148 (98, 231)^b^	103 (0, 330)
Snack kcal [Mean (min, max)]	185 (90, 620)	198 (198, 198)^c^	294 (142, 482)	148 (10, 213)	197 (36, 528)	198 (10, 740)

*Note.* Site 2 had 394 temporary agency staff and Site 6 had additional 476 agency staff for which they did not have demographic information and are therefore not included in this Table. The mean values denoted in the sales data reflect averages of actual items sold. ^a^Energy estimates for drinks at Site 3 are expressed per 100 ml. ^b^Site 5 does not sell low calorie soft drinks (this site produces soft drinks which are freely available to all staff, including sugar-free and sugar-sweetened varieties). ^c^Site 2 offered only a limited selection of snacks sold under the same till button, hence the median energy value was used to estimate energy content of snacks at this site

### Design and procedure

**Fig. 2:**
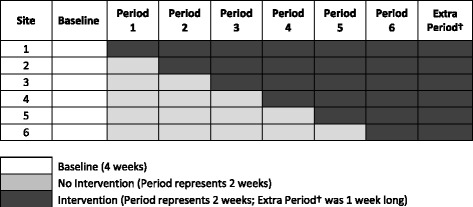
A graphical presentation of the study’s stepped wedge design

The study was a stepped wedge randomised controlled trial [13]. This can also be described as a staggered interrupted time series design [14]. Six worksite cafeterias were sequentially randomised to receive the intervention after an initial baseline period of 4 weeks (see Fig. 2). Within each of the six worksite cafeterias, the time at which the intervention was introduced was randomly allocated by means of random permutations using random variates of the uniform distribution to mitigate for possible confounding time effects whilst maximising sample size [15]. The randomisation and assignment of sites to the intervention sequence was performed by a statistician (DLC) using computer software. A sample size of six sites was selected prior to enrolment as a pragmatic number with which to assess feasibility.

In all six worksite cafeterias all sales were recorded as part of a baseline period of at least 4 weeks. Sites were then randomised to implement the intervention at one of six, two-weekly intervals. An extra intervention week at the end of the trial was captured for all six sites resulting in a total of 13 intervention weeks. Once randomised to the intervention, each site maintained the intervention until the end of the study, i.e. the period of intervention in the sites varied from between three and 13 weeks. Patrons of the worksite cafeterias were not informed that the study was taking place, but the cafeteria caterers who implemented the intervention were not blind to the intervention. Caterers were trained and instructed on how to implement the intervention prior to the trial start date. Data on the energy content of food and drink items was supplied by the sites, and data on sales was obtained from the sites’ till records.

### Intervention

The intervention comprised labelling all cafeteria products for which calorie information was available with their energy content (e.g.*,* “250 Calories” or “250 Cals”). Calories were denoted in the same font style and size as used for the product name or price, whichever was the largest (detailed labelling guidelines sent to the sites prior to the introduction of the intervention can be seen in Additional file 1). The labels were intended to be legible and prominent to the customer from where they were standing at the point of choice. The portion size of the food items labelled was made clear using such additions as ‘per slice’, ‘per ladle’, or ‘per average bowl/serving’ if they were pre-portioned or served to the customer. Some sites chose to also include similar information presented in kJ. Salad bars, hot drinks, and vending machine items were excluded from the intervention (for more details see Additional file 1).

**Fig. 3:**
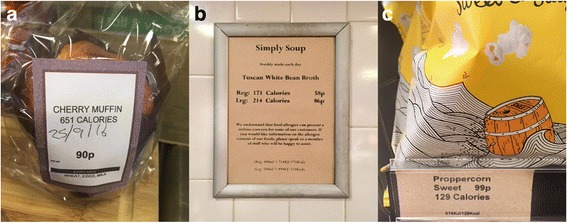
Examples of calorie labelling: **a**) on a product, **b**) on a menu; and **c**) along shelf-edging at point of choice

Calorie information was provided in one of three places:

1. On products (printed or hand written; see Fig. 3a);
2. On menus (printed or electronic via email or screens; see Fig. 3b);
3. Along shelf edging at point of choice (see Fig. 3c).

### Measures

#### Feasibility and acceptability

The *feasibility of recruiting and retaining eligible worksites* was assessed by recording recruitment rates, and the number of worksite cafeterias dropping-out of the pilot trial i. during the baseline period, ii. during the intervention period, or iii. post-recruitment. The *feasibility of implementing the assigned intervention* was assessed by the research team after initial visits to worksite cafeterias, discussions regarding suitability with worksite managers and catering teams, and by examining pre-intervention sales data supplied by the sites. Qualitative interviews with worksite managers provided an additional measure of potential challenges with implementation of the intervention.

We gauged the *acceptability of the intervention* by surveying patrons of the worksite cafeterias. All employees based at the six worksites were invited to complete an anonymous survey distributed through an all staff e-mail containing a link to the survey (five sites), or by handing out paper copies within the cafeteria (one site). Qualitative interviews with worksite managers/caterers supplemented the survey data, providing insight into the acceptability of the intervention and the assessment procedures.

We evaluated *compliance with the study protocol* during compliance visits coinciding with the initial period of intervention implementation for each randomised worksite. For each worksite the research team conducted one compliance visit in the first intervention week. Subsequent compliance was recorded by the sites sending the research team photographs of the cafeteria each week.

#### Intervention impact

The published protocol delineates the analyses planned to estimate the impact of the labelling intervention [11]. The unit of analysis was the worksite cafeteria per day, and not the individuals using the worksite cafeteria as only aggregate till transaction data were available.

Some changes were, however, made to the pre-registration analysis plan as none of the sites could provide calorie information for non-intervention items (e.g.*,* salad bars, deli bars, hot drinks, and vending machine items; for more information see Additional file 1), thus necessitating the analysis of total energy purchased only from intervention items for the primary outcome, and modifying the secondary outcome to examine the number of items purchased from intervention and non-intervention items. The outcomes assessed were as follows:

##### Primary outcome

Total energy (kcal) purchased per time frame of analysis (daily) from intervention items, controlling for the total transactions as measured from daily sales records.

##### Secondary outcome

Number of items purchased per time frame of analysis (daily) from (a) intervention items, and (b) non-intervention items, controlling for the total transactions.

##### Other measures

Covariates measured in the study were: worksite demographic characteristics (see Table 1); day of week; and weather conditions (daily average temperature). Worksite demographic characteristics and daily average temperature could not be fitted in the final model as this would have led to overfitting.

### Data analysis

#### Feasibility and acceptability

Descriptive statistics were used to summarise feasibility and acceptability outcomes, including recruitment and attrition rates. Qualitative assessments gathered via semi-structured interviews with worksite cafeteria staff were coded and summarised in narrative form.

#### Intervention impact

Analyses were conducted in R.3.3.3. Linear models were used to estimate the potential impact of the intervention and associated effect sizes. Generalised linear mixed models (GLMM) were fitted to examine the impact on total energy (kcal) purchased per day from intervention items controlling for the total transactions, adjusted for time trends (using day relative to the intervention start date as a random slope per site) and with random effects for worksite.

Initial analyses were conducted to confirm the selected time frame level of daily (as opposed to weekly) sales due to day-level events that occurred irregularly during the study period affecting sales in cafeterias such as corporate training events. Analysing the data at the week-level would have biased the estimates by not taking into account unexpected events across time at the different sites. A daily time frame was also selected since the conditional distribution of the daily outcome was compatible with the model assumptions and, since there were no time-dependencies present in the data after controlling for total transactions, time and other variables. Uncharacteristic days – such as days showing large changes in energy purchased due to corporate events - were included as dummy variables to allow for an unbiased estimate of the intervention effect (see Results for more details).

It was not appropriate to attempt to exclude outliers, as the non-stationary nature of the data meant that many arbitrary assumptions would have been required in defining outliers. The intervention was fitted as six dummy fixed effects for each site. Site was fitted as a random effect, with random intercept and random gradient for day i.e. day number relative to the intervention – day 0 being the day of implementation.

The unit of analysis was the worksite cafeteria per day, not the individuals using the worksite cafeteria as only aggregate till transaction data were available. This gave less power than originally anticipated in the protocol. Sensitivity analysis was conducted to explore whether partial compliance with the intervention affected the obtained results.

## Results

### Feasibility and acceptability

Of the 39 worksites approached, 18 were eligible and all 18 agreed to take part. Of the 18 eligible worksites six sites received the labelling intervention (with the 12 remaining worksites randomised to receive the availability and size interventions, see published protocol [11]). All six worksite cafeterias recruited for the intervention successfully completed the baseline and intervention periods (attrition rate of 0%), attesting to the feasibility of recruiting and retaining eligible worksites (see also Fig. 1 for the flow of participating sites throughout the study).

The feasibility of implementing the intervention varied greatly between sites, with the proportion of items suitable for labelling ranging from 50% to 99%. In addition, for the items that could be labelled, several barriers to implementation were identified. First, the energy estimates of the cooked meals provided by the sites may have been under- or over-estimated depending on the recipes used. We checked these estimates by comparing the energy content of randomly selected non-packaged items to energy estimates from three published recipes matching the item description. In addition, the precision of the energy estimates of the cooked meals may have been compromised due to meals at some sites being prepared “at the chef’s discretion” in using recipes. In the present study we were not able to verify whether the energy estimates provided by the chefs matched their execution of the recipes. Second, the precision of sales data was variable. In one site, all the sales data were manually recorded, and a few other sites sold multiple items under the same till button. The site that manually recorded the data used their stock records to validate the data, and for the few items where the same till button was used to record sales we used the median energy estimate of all items captured under the single button.

The acceptability of the intervention was generally high amongst those who took part in the post-study survey. Very few employees participated in the survey (*n* = 192, approximately 4% of the total number of employees based at the six sites). Participants were asked “*How did you feel about the introduction of calorie labels?”* (rated on a five-point scale from *Very displeased* to *Very pleased*, with an additional option of choosing *Didn’t notice the labels*). Of those who completed the survey, 63% were either pleased or very pleased about the introduction of calorie labelling, 18.8% were neither pleased nor displeased, 0.5% were displeased or very displeased, whilst 17.7% reported not noticing the changes in labelling. Participants also responded to the question *“Would you like calorie labels to remain in place permanently?”* (rated on a five-point scale from *No, definitely not* to *Yes, definitely*). The majority (74%) self-reported they would like calorie labelling to remain in place permanently (answering either *Yes, definitely* or *Yes, probably*), 22.9% didn’t mind, whilst only 3.1% objected to calorie labelling remaining in place permanently (answering either *No, probably not* or *No, definitely not*). Similarly high levels of acceptability were reported amongst worksite managers during their post-intervention interviews. We carried out a thematic analysis on the points raised by the worksite managers (see Table 2 for more details on the themes and sub-themes identified). Overall, worksite managers were receptive and highly supportive of the intervention seeing it as adding rather than taking something away from patrons. They commented that the initial implementation of calorie labelling was labour-intensive and time-consuming due to the gathering of information and production of calorie labelling. However, once this was done, the intervention was easy to maintain. The managers were also gratified to note the absence of negative feedback and the high acceptability of the intervention expressed by the cafeteria patrons.

**Table 2:** Themes identified from semi-structured interviews with worksite managers

Themes	Sub Themes	Comments
Information Provided	Clear information (5 of 6 Sites)	*“Labels were clear and easy to understand.”*
	Concise information (2 of 6 Sites)	*“Really good, clear and quality labelling.”*
	Missing information (3 of 6 Sites)	*“We used to list fat, salt and sugar… I think sometimes people just get a bit confused by it all”*
		*“People started to ask about salt, started to ask about sugar…”*
Implementation Issues	Issues in getting calorie information (4 of 6 Sites)	*“What had always stopped us in the past was the chefs and their freedom to be chefs”*
	Time-consuming to implement (6 of 6 sites)	*“The blocker for us is getting the calories of the meals that they make on site and being accurate with that”*
	Easy to run (6 of 6 sites)	*“ There is a lot of sending data which is very time consuming”*
		*“Once you just get on with it and when you start to understand, actually its fine”*
Feedback from Customers	Addition of information (5 of 6 sites)	*“Because we were adding information for people and not taking anything away from them, I think that was a positive thing”*
	Awareness of labels (6 of 6 sites)	*“…it was noticed and people were pleased to see it. They were surprised actually and it was driving them to make difficult choices.”*
		*“Nobody really commented on it either way… it certainly wasn’t an issue.”*

Compliance with the study protocol varied across sites. Compliance visits were conducted at each site during the first week of intervention when non-compliant items, i.e. unlabelled products, were noted. Additional file 2: Table S1 provides a detailed breakdown of non-compliant items per site and denotes the date when unlabelled items were labelled as per protocol in each site. Sensitivity analyses were performed to check whether there were significant differences in the effects of the intervention between days when all items that could be intervened upon were labelled and days when they were not.

### Intervention impact

**Fig. 4:**
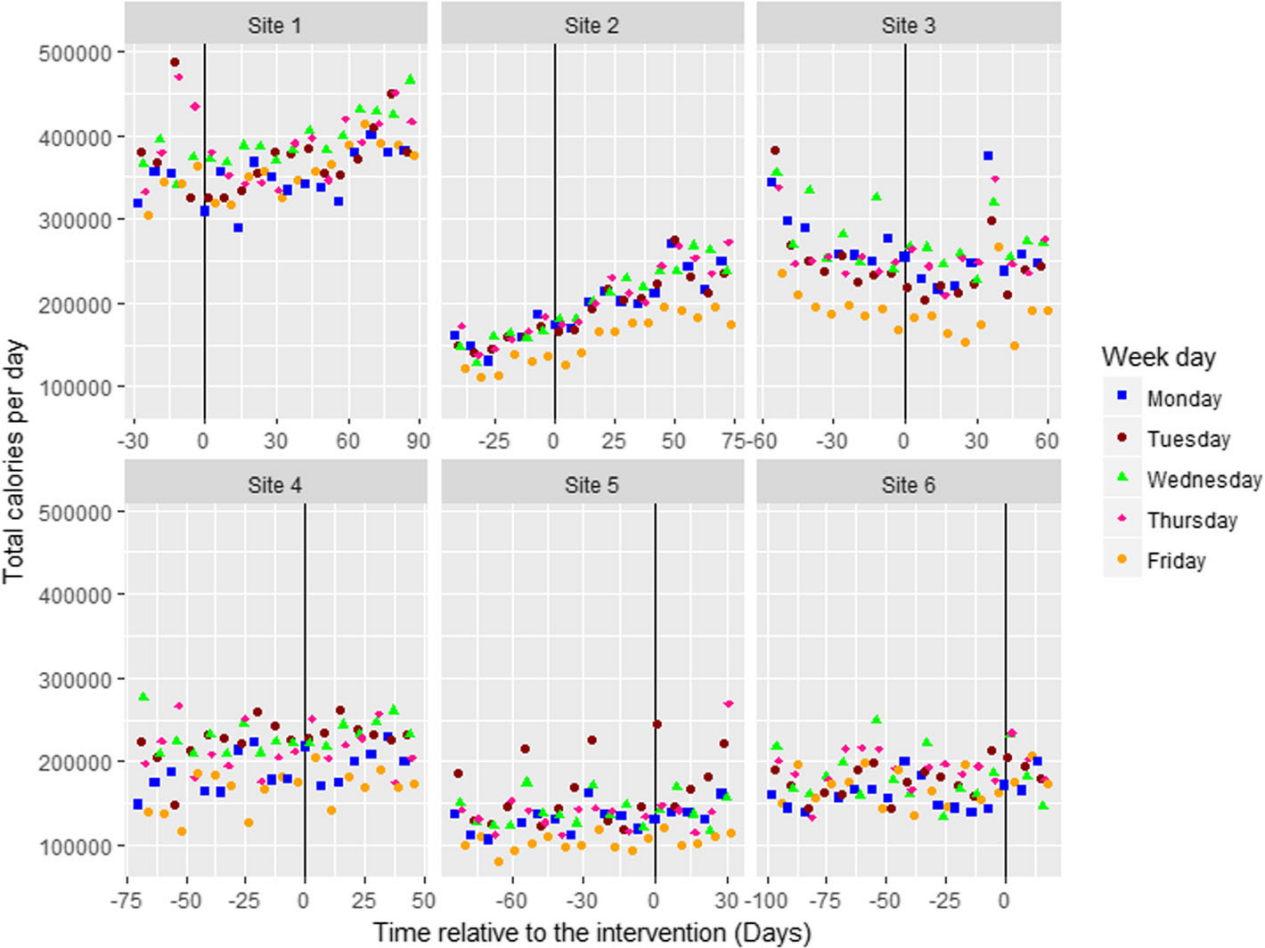
Total energy sold per day for intervention items across the six sites with information displayed for day of the week

The data were collected from six sites over 116 days (8th August to 2nd December 2016). The total energy purchased from intervention items per day and week at each site varied widely, revealing different underlying trends at different sites. As can be seen in Fig. 4, graphically presenting the total energy sold at each site revealed the following: (i) time trends in the data, varying by site and by pre- and post-intervention, which had to be accommodated; (ii) strong weekday effects (e.g.*,* with less energy purchased on Fridays); and (iii) regular features in some of the sites that had to be accounted for by dummy variables. For example, at Site 1 there were three staff training days just before the intervention, when many more people used the restaurant; whilst at Site 3 there was a week pre-intervention and a week post-intervention where external people attended meetings, inflating the total daily calories purchased. Finally, at Site 5 there was a regular event on the first Tuesday of almost every month causing an almost doubling of the daily total calories purchased. Although management were unable to determine the cause of this, it was too regular an occurrence to have happened by chance.

**Fig. 5:**
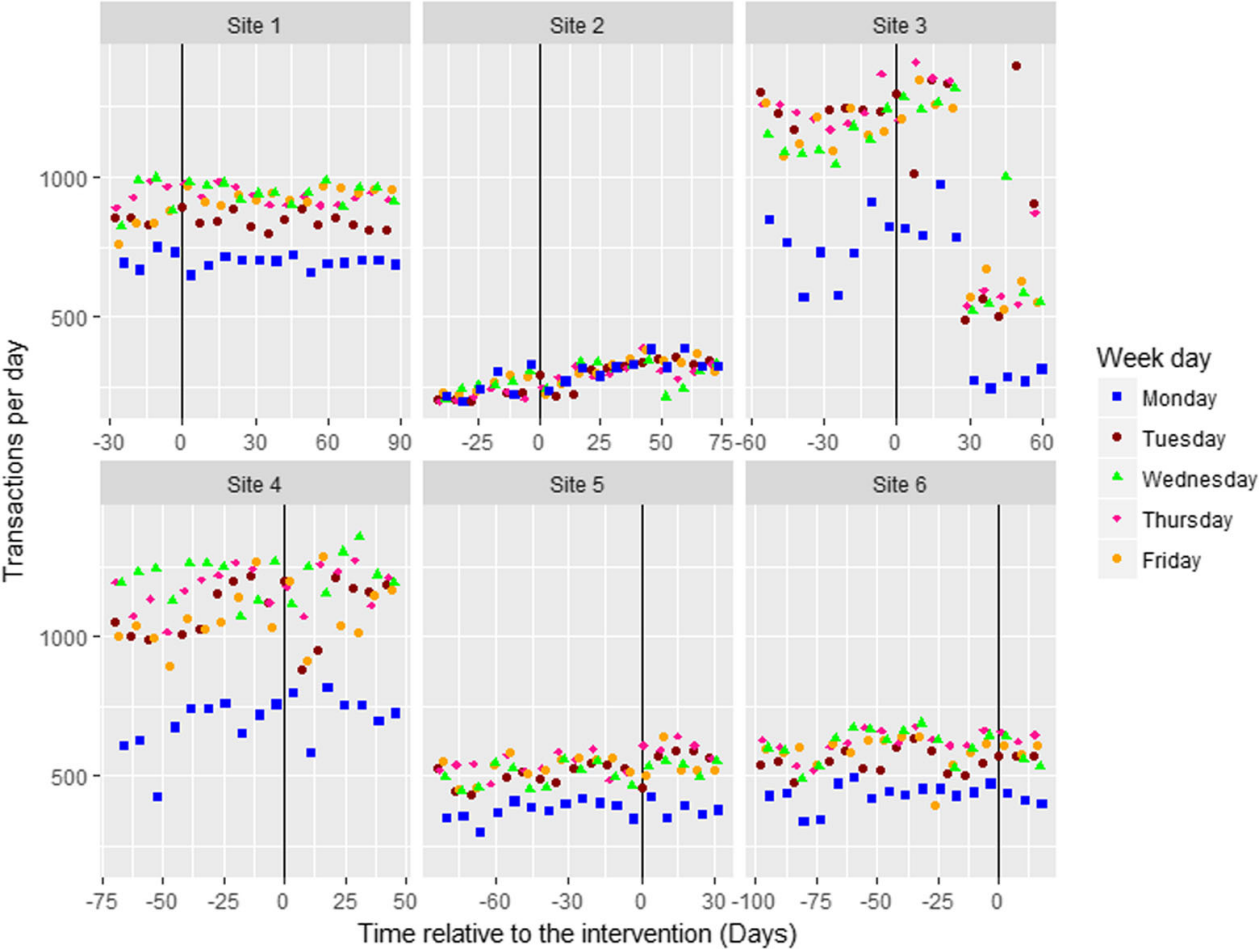
Transactions per day for intervention items across the six sites with information displayed for day of the week

Furthermore, as can be seen in the graphical presentation of the transaction data (Fig. 5), at Site 3 the total number of transactions appeared to fall markedly. Investigations suggested that this was due to a change in processing, when a “group transaction” till was introduced on certain days. As transactions were used as a controlling covariate in the primary analysis, this feature also required modelling using a dummy variable.

Given the small sample size, there was limited scope to include explanatory terms in the modelling. The final model included number of transactions, time relative to the intervention, week-day and the five unusual features across the six sites as covariates. Model diagnostics (i.e.*,* residual plots, autocorrelation) were acceptable. Alternative models were also examined (see sensitivity analysis below).

### Primary outcome

There was no overall effect of the intervention: -0.4% (95%CI -3.8 to 2.9, *p* = .803, *M* = − 916.5 (*SD* = 3667) total daily calories). One site (Site 1) showed a statistically significant effect of the intervention, with an estimated 6.6% reduction (95%CI -12.9 to − 0.3, *p* = .044) in energy purchased in the day following the introduction of calorie labelling, an effect that diminished over the study period. This was calculated using pre-intervention average daily energy purchases per site as the denominator, and the percentage change was robust to choosing different pre-intervention periods. A linear model fitted to the post-intervention data at Site 1 estimated it would take approximately 60 days (8.6 weeks) for energy purchases to return to the pre-intervention daily average (see Table 3). The remaining sites did not show robust changes in energy purchased when calorie labelling was introduced.

**Table 3:** Primary analysis of total daily energy purchased

Fixed effects	Calories *M (SD)*	95%CI	p	Pre-Intervention Mean Daily Calories	% Change Post-Intervention	95%CI
(Intercept)	150637.1 (28779.4)	(94230.4, 207043.7)	< 0.0001			
Site 3 Till Change^b^	71573.5 (11394.4)	(49240.8, 93906.1)	< 0.0001			
Site 3End Outliers^b^	88118.6 (11036.5)	(66487.5, 109749.6)	< 0.0001			
Site 3Start Outliers^b^	75880.6 (12624.4)	(51137.2, 100624.0)	< 0.0001			
Site 5 Outliers^b^	90157.4 (10249.5)	(70068.7, 110246.1)	< 0.0001			
Site 1 Outliers^b^	94505.4 (14167.9)	(66736.9, 122273.9)	< 0.0001			
Week day (Ref = Monday)						
Tuesday	7960.1 (3177.8)	(1731.8, 14188.4)	0.0150			
Wednesday	21430.7 (3125.8)	(15304.4, 27557.1)	< 0.0001			
Thursday	12899.7 (3148.5)	(6728.8, 19070.7)	0.0001			
Friday	− 9580.2 (3807.9)	(−17043.5, − 2116.8)	0.0146			
Transactions	70.1 (10.1)	(50.3, 89.8)	< 0.0001			
Intervention^a^						
Site1	−24381.2 (11893.4)	(−47691.7, − 1070.6)	0.0448	370114.3	−6.6%	(−12.9%, −0.3%)
Site2	1742.0 (10962.2)	(−19743.5, 23227.4)	0.8743	150105.7	1.2%	(−13.2%, 15.5%)
Site3	2737.8 (10968.1)	(−18759.3, 24234.9)	0.8038	256231.0	1.1%	(−7.3%, 9.5%)
Site4	945.7 (10962.4)	(−20540.1, 22431.5)	0.9315	200117.1	0.5%	(−10.3%, 11.2%)
Site5	12407.6 (10962.0)	(− 9077.6, 33892.7)	0.2623	131628.9	9.4%	(−6.9%, 25.7%)
Site6	14836.6 (10841.4)	(− 6412.1, 36085.3)	0.1763	174700.4	8.5%	(−3.7%, 20.7%)

*Note*. *P*-values are based on the Kenward-Roger correction to DF. ^a^Site-specific intervention effects were obtained by refitting the model with a different reference category. ^b^Dummy variables for outliers

### Sensitivity analysis

To avoid overfitting, only limited additional explanatory variables could be considered in sensitivity analyses. A sensitivity analysis was conducted in which all items for which there was only partial calorie labelling at each site at any point during the intervention phase, were excluded when calculating the total calories per day. This led to the removal of 21 (6.5%), 19 (25.3%), 5 (1.2%), 26 (35.6%), 34 (21.1%) and 159 (30%) products at Sites 1–6 respectively. Similar results were obtained to those obtained using the primary models. There was no overall effect of the intervention [221 (0.1%), 95%CI -6527 to 6971 (− 3.7% to 3.9%), *p* = .949], with a significant reduction in energy purchased in intervention items at Site 1 [− 26290 (− 7.1%), 95%CI -48382 to − 4197 (− 13% to − 1.1%), *p* = .024].

### Secondary outcome

As it was not possible to model the total daily energy for non-intervention items separately, we considered total number of (a) intervention items and (b) non-intervention items sold per day, as an alternative secondary outcome to gauge whether the calorie labelling intervention also affected sales of non-intervention items.

#### All items (combining both intervention and non-intervention items)

Using transactions as a covariate, there was no overall effect of the intervention on total sales of all items per day (including both intervention and non-intervention items) (− 16.0 items (− 1.9%), 95%CI -62.3 to 30.2 (− 7.4% to 3.6%), *p* = .514). There were also no significant results at individual sites.

#### Intervention items only

Using transactions as a covariate, there was no overall effect of labelling on total sales of intervention items per day [− 10.2 items (− 0.9%), 95%CI -48.3 to 28.0 (− 4.1% to 2.4%), *p* = .618]. There was only one significant effect at Site 1 [− 233.2 (− 11.8%), 95%CI -359.7 to − 106.8 (− 18.2% to − 5.4%), *p* = .009]. A linear model fitted to the post-intervention data at Site 1 showed it took approximately 49 days (7 weeks) to return to the pre-intervention sales rate.

#### Non-intervention items only

Using transactions as a covariate, there was an overall effect of the intervention on total sales of non-intervention items per day [− 23.9 items (− 3.2%), 95%CI -44.0 to − 3.9 (− 5.9% to − 0.5%), *p* = .024]. There were two significant effects at Site 3 [− 83.3 (− 9.9%), 95%CI -143.2 to − 23.4 (− 17% to − 2.8%), *p* = .015], and Site 5 [65.3 (13.4%), 95%CI 5.5 to 125.2 (1.1% to 25.8%), *p* = .048]*.* A linear model fitted to the post-intervention data at Site 3 showed it took approximately 4 days (0.6 weeks) to return to the pre-intervention sales rate. For Site 5, the linear model predicted a constant increase in sales. Caution should be taken in interpreting all these statistical results, due to the small sample size and considerable sources of variability.

## Discussion

The recruitment of six sites for the pilot trial proved feasible. The conduct of the study was also feasible, with no sites dropping out of the intervention. Post-intervention feedback amongst cafeteria patrons and worksite managers and caterers suggested high levels of acceptability. There was no overall effect of calorie labelling upon energy purchased across six worksite cafeterias. A statistically significant reduction in total calories purchased from intervention items was apparent at Site 1, with an estimated 6.6% reduction, though this effect diminished over time returning to pre-intervention levels after approximately 60 days. The remaining five sites did not show robust changes in energy purchased when calorie labelling was introduced. We discuss first the estimated impact of the calorie labelling intervention and how it fits with prior empirical work before providing more details about the implementation of the intervention.

The direction and the size of the effect at Site 1 fits recent systematic review evidence showing that calorie labelling has the potential to reduce the amount of energy purchased [10]. The lack of a significant reduction in energy purchased in five out of the six worksites may have several explanations relating to barriers to intervention implementation, the nature of the intervention, and site differences. Accordingly, the present findings extend the mixed outcomes from synthesized evidence regarding different choice architecture interventions in worksites [9], by showing that calorie labelling may work only in certain settings.

The barriers to effective intervention implementation that could explain the lack of significant effects in five of the six sites, include the differential ability to intervene upon cafeteria products, chef’s discretion at implementing cooked meal recipes, till button issues with recording sales data, and freely available foodstuffs in the cafeterias and elsewhere across the worksites. Sites differed in the proportion of cafeteria products that were labelled (50–99%), due to availability of energy information. Due to the small sample size and resulting degrees of freedom in analyses, we could not model the moderating effects of partial implementation on the effectiveness of the labelling intervention, however future studies may explore whether the success of the intervention is proportional to the level of intervention implementation.

In four of the six sites (Sites 2, 4, 5 and 6) there were a few items that were recorded under the same till button (e.g.*,* sales of different fizzy drinks recorded under the same till button). Since the different food/drink items may have had different energy content, we calculated the median energy content for the sales records of these till buttons. Since this was not an issue that affected many of the sold items, this should not have unduly influenced the statistical analyses, however it is possible that some of the intervention effect has been masked if energy labelling swayed cafeteria patrons from one item to another represented by the same till button. Catering managers of the six sites identified chef’s discretion at implementing meal recipes as an additional barrier to intervention implementation. While this would have affected the accurate estimation of the energy content of food it would be expected to be similar pre- and post-intervention, and should not have unduly affected our results.

Foodstuffs such as confectionery and fruits were available free to employees across the six worksites. Since the sites keep no record of the availability and intake of these freely available foodstuffs, we could not control for this factor in our analyses. Future studies should examine different ways of controlling for this extraneous variable.

Calorie labels were designed to be prominent to the customer at the point of choice, and were presented in the same font style and size as the product price. These features may have also inadvertently decreased the impact of the intervention by making the calorie information less distinguishable from all the other information on the product label, thus dampening the potential impact of calorie labelling. Further studies, including laboratory experiments, are needed to elucidate whether design features of the labels could boost the effectiveness of the intervention.

In addition, the demographic characteristics of the employees across the six sites varied widely which could explain the differences in pre- and post-intervention patterns in food purchasing across the six sites. Due to the small sample size in this pilot trial we were not able to examine the potential moderating effects of the sites’ demographic composition on calorie labelling. Future work of this nature should aim to increase the sample size to allow the testing of moderation effects. Furthermore, a larger sample size may have helped disentangle the intervention effect from possible time-confounding effects, which is a known limitation of stepped wedge designs [16].

### Strengths and limitations

This pilot trial is one of the first to measure the impact of calorie labelling across multiple sites over a relatively extended time-period. As such, it is one of the largest studies to date to examine how calorie labelling affects purchasing in worksite cafeterias (see [9]). A further strength of the current study is the use of a stepped wedge design which combines features of within- and between-subjects designs thus allowing the examination of changes in purchasing within each worksite cafeteria depending on the time-period (pre- vs. post-intervention), whilst at the same time allowing examination of differences in purchasing between worksites at different time-points.

The above strengths notwithstanding, this study was limited in several respects. The present study sampled food and grocery industry worksites that wanted to encourage healthier eating amongst their workforce, and as such this may not be a fully representative sample of worksites. Sites were able to provide only aggregate sales data at site level, rather than sales for each individual worker. Additional individual-level data would have provided more power to allow more parameterised models to be fitted. Furthermore, purchasing rather than consumption of energy was the study outcome. This outcome may not reflect the actual consumption of the cafeteria patrons, since food obtained from other sources or food waste is not taken into account. Moreover, the energy content of non-intervention items could not be estimated accurately, thereby precluding the estimation of total energy purchased including both intervention and non-intervention items. Other limitations pertain to the duration of the baseline period as well as the difficulty with modelling the influence of the time-trend within the stepped wedge with a relatively small number of sites. For example, a longer baseline period would have allowed estimates of whether the total daily energy sold was in a steady state pre-intervention. In addition, the unexpected events caused noise within the data making it more difficult to detect possible intervention effects.

### Implications for research and policy

This pilot trial has identified some significant and complex challenges in estimating the effect of calorie labelling in real-world worksite settings. The present findings warrant further research addressing the main possible explanations for a weak or null effect of calorie labelling on sales. Such research includes evaluating the impact of more vivid calorie labels, addressing the barriers to implementation identified, and increasing the number of observations whilst decreasing variability within and between sites. In terms of policy implications, the present findings suggest that calorie labelling may, in certain settings, be effective in reducing the total energy purchased in worksite cafeterias. However, more research is needed to elucidate the boundary conditions that make calorie labelling more or less effective in different settings which might include the socio-demographic composition of worksite employees, prior motivations of employees to reduce energy consumption, as well as any other health initiatives implemented by the worksites.

## Conclusions

A calorie labelling intervention was acceptable to both cafeteria operators and customers. The predicted effect of labelling to reduce energy purchased was only evident at one out of six sites studied. Before progressing to a full trial, the calorie labelling intervention needs to be optimised, and a number of operational issues resolved.

## Additional files

Additional file 1: Calorie Labelling Guidance. (PDF 208 kb)

Additional file 2: **Table S1.** Compliance with study protocol per site. (PDF 141 kb)
