# Supplementary material for: Impact of calorie labelling in worksite cafeterias: a stepped wedge randomised controlled pilot trial
Source: Int J Behav Nutr Phys Act. 2018 May 14;15:41. doi: 10.1186/s12966-018-0671-7 (PMC5950179; doi:10.1186/s12966-018-0671-7)
Supplement: Supplementary file 1 — Calorie Labelling Guidance. (PDF 208 kb) [file 12966_2018_671_MOESM1_ESM.pdf]

Additional File 1: Calorie Labelling Guidance

Impact of calorie labelling in worksite cafeterias: A stepped wedge randomised controlled pilot trial

Milica Vasiljevic[1], Emma Cartwright[1], Mark Pilling[1], Mei-Man Lee[2], Giacomo Bignardi[1], Rachel Pechey[1], Gareth J. Hollands[1], Susan A. Jebb[1,2], & Theresa M. Marteau[1]

[1] Behaviour and Health Research Unit, Institute of Public Health, University of Cambridge, Cambridge, UK

[2] Nuffield Department of Primary Care Health Sciences, University of Oxford, Oxford, UK

Calorie Labelling Guidance

As part of your participation in IGD's Healthy Eating in the workplace pilots, your site will be taking part in the Calorie labelling intervention. To ensure the research is a success we ask you to follow the protocol you have been sent, and from your specified date, display Calories for all products at point of choice, following the below guidance.

Allergen information should continue to be provided as usual.

Label Design:

- Name of food or drink item
- Calorie content written as 'XXX Calories' or 'XXX Cals'
- Calorie labels should be:
  - same font type/style as the product name
  - same font size as the product name/price (whichever is the biggest)
  - Legible and prominent to the customer (from where they will be standing at point of choice.)
  - Ideally rounded to the nearest 5 or 10 Calories
  - Portion size must be clear (e.g. per slice, per ladle, per average bowl/serving if pre-portioned or served to the customer)
  - Any additional information such as Reference Intakes should be removed
  - KJ and Kcal information can be displayed at the bottom of the label, this must be in a smaller font to the Calories information, see figure 1 for an example

Please find some examples below.

What products to label

All products within the cafeteria should be labelled with Calorie information, including:

- Main meals (including side dishes)
- Snacks (including all confectionary, sandwiches, protein pots etc.)
- Breakfast selection (both hot and cold selection)
- Cold Drinks and snacks (even if prepacked with nutrition information)
- Condiments (portioned)

Salad bars, hot drinks and vending are **excluded** from the study and therefore do not require labels (however, please note you will still need to send daily sales information for salad bars to UoC).
Other anomalies such as deli bars will be dealt with case by case by the University of Cambridge

Where to put labels:

Calorie information should be directly above, below or beside the product. Where this is not possible inform the UoC to discuss.

- Menus (printed or electronic via email or screens)
- Labels on products (printed or hand written)
- Shelf edging at point of choice
- Tent cards next to products

Examples of Labels: Figure 1:

Baked Beans

110 Calories

(per serving)

(460KJ/110Kcal)

Croissant

£1.00

260 Cals

(1040KJ/260Kcal)

Examples of product labels: Figure 2:

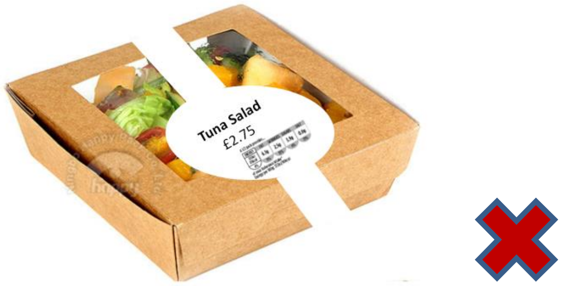

This is not correct as full Reference Intake (RI) information is displayed instead of Calories.

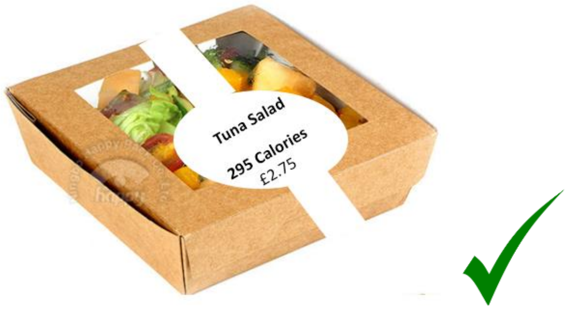

This is correct.
