# Supplementary material for: Impact of calorie labelling in worksite cafeterias: a stepped wedge randomised controlled pilot trial
Source: Int J Behav Nutr Phys Act. 2018 May 14;15:41. doi: 10.1186/s12966-018-0671-7 (PMC5950179; doi:10.1186/s12966-018-0671-7)
Supplement: Supplementary file 2 — Table S1. Compliance with study protocol per site. (PDF 141 kb) [file 12966_2018_671_MOESM2_ESM.pdf]

Additional File 2: Table S1. Compliance with study protocol per site

Impact of calorie labelling in worksite cafeterias: A stepped wedge randomised controlled pilot trial

Milica Vasiljevic[1], Emma Cartwright[1], Mark Pilling[1], Mei-Man Lee[2], Giacomo Bignardi[1], Rachel Pechey[1], Gareth J. Hollands[1], Susan A. Jebb[1,2], & Theresa M. Marteau[1]

[1] Behaviour and Health Research Unit, Institute of Public Health, University of Cambridge, Cambridge, UK

[2] Nuffield Department of Primary Care Health Sciences, University of Oxford, Oxford, UK

Table S1.

Compliance with study protocol per site.

**Site**	**Intervention Start Date**	**Compliance Visit Complete**	**Missing Information**	**Location**	**Confirmed by Site**
**Site 1**	05/09/2016	06/09/2016	Yoghurt Pot	Chiller	07/09/2016
			Milk 189ml, Milk 250ml and Milk Pint	Chiller	07/09/2016
			Tropicana	Chiller	07/09/2016
			Mousse	Chiller	15/09/2016
			Jacket Potato, Jacket Potato Topping and Jacket Potato with Hot Filling	Hot Food	07/09/2016
			Apple, Banana	Till point	07/09/2016
			Cake	Till point	15/09/2016
**Site 2**	19/09/2016	20/09/2016	All drinks: (Coke (139 Kcal), Diet coke (<1 Kcal), Cherry coke (149 Kcal), Coke Zero (0 Kcal), Fanta Orange (87 Kcal), Fanta Lemon (112 Kcal), Sprite (144 Kcal), Dr. Pepper (96 Kcal), Coke (210 Kcal), Diet coke (<1), Cherry coke (225 Kcal), Coke Zero (0 Kcal), Fanta Orange (140 Kcal), Fanta Lemon (169 Kcal), Sprite (135 Kcal), Dr. Pepper (145 Kcal), Orange fruit juice)	Drinks chiller	23/09/2016

			Golden Wonder crisps	Till point	23/09/2016
			Real crisps	Till point	23/09/2016
			Chocolate bars	Till point	23/09/2016
**Site 3**	03/10/2016	05/10/2016	Popcorn, Kettle Crisps 100g	Chillers	11/10/2016
			Soft Drinks_Pepsi, 7Up, Drench, 100ml Labelled per 100ml rather than full bottle	Chillers	11/10/2016
			Orange, Apple, Cranberry 100ml, Ribena	Chillers	11/10/2016
**Site 4**	17/10/2016	18/10/2016	No labels on pre-packaged items (canned soft drinks, confectionery & gum, Copella, Deli Crisps, Eat Natural bars, Go Ahead Bar, Graze Box, Innocent Smoothie, Joe & Seph popcorn, Juicy water, Kit Kat 4 finger, Oasis, Penn State pretzels, Popchips, Propercorn, rice cakes, Salty Dog crisps, sesame snaps, snack bags, snack essential, Tropicana juice, Walkers crisps	Café and main restaurant	A4 lists displayed on 24/10/2016, range labels displayed on 31/10/2016
			Labelling on items not consistent (Sandwich - £2.50, sandwich - £3.25, sandwich - £3.50, sandwich	Sandwiches/salads/fruit pots	Labels formatted correctly on 24/10/2016

			£1.95, yoghurt/fruit pots/ granola)		
			Calorie information on menus in smaller font than the price (£3.38 Bistro, £3.60 Bistro, £4.00 Bistro)	Main menus	24/10/2016
		2nd Visit completed 02/11/2016	Swaps signage displayed (a Sodexo incentive) – displayed on the 31st Oct onwards	Signage on the floor of the restaurant and A4 signage on top of the hot counters	Removed – from 10/11/2016
**Site 5**	31/10/2016	02/11/2016	Confectionery_Crisps and Snacks (all 14 items)	Till points and chillers	18/11/2016
			Retail Sandwich_Delifresh (all 8 fillings)	Chiller	18/11/2016
			Fresh fruit	Chiller	18/11/2016
**Site 6**	14/11/2016	15/11/2016	Pre-Packaged items (crisps, chocolate, drinks etc.). A4 lists displayed but no individual product labels	Café and Restaurant	18/11/2016
